# Inverse association between the anticholinergic burden and hippocampus volume in a population-based cohort across the entire adult age range

**DOI:** 10.1007/s11357-021-00497-w

**Published:** 2021-12-23

**Authors:** Ingo Kilimann, Diana Wucherer, Till Ittermann, Henry Völzke, Robin Bülow, Wolfgang Hoffmann, Hans Jörgen Grabe, Katharina Wittfeld, Stefan Johannes Teipel

**Affiliations:** 1 German Center for Neurodegenerative Disease (DZNE), Rostock/Greifswald, Gehlsheimer Straße 20, 18147 Rostock, Germany; 2 Department Psychosomatic Medicine and Psychotherapy, Rostock University Medical Center, Gehlsheimer Straße 20, 18147 Rostock, Germany; 3 German Center for Neurodegenerative Disease (DZNE), Rostock/Greifswald, Ellernholzstraße 1-2, 17475 Greifswald, Germany; 4 SHIP Study Unit, Institute for Community Medicine, University Medicine Greifswald, Walther-Rathenau-Str.48, 17475 Greifswald, Germany; 5 Institute of Diagnostic Radiology and Neuroradiology, University Medicine Greifswald, Ferdinand-Sauerbruch-Straße, 17475 Greifswald, Germany; 6 Institute for Community Medicine, University Medicine Greifswald, Ellernholzstraße 1-2, 17475 Greifswald, Germany; 7 Department of Psychiatry and Psychotherapy, University Medicine Greifswald, Ellernholzstraße 1-2, 17475 Greifswald, Germany

**Keywords:** Cognitive impairment, Hippocampus, Medication, Alzheimer’s disease

## Abstract

Many medications of different indications have a relevant anticholinergic activity. The anticholinergic burden of medication has been shown to have significant effects on the cognition and the risk for cognitive impairment and dementia particularly in older patients. So far, most of the studies used data from geriatric patients and the effect of the anticholinergic burden on brain structures is still unexplored. Our study aimed to analyze possible associations of hippocampus and cholinergic basal forebrain volumes as vulnerable brain structures for the development of dementia and the anticholinergic burden in a population-based cohort of non-demented participants spanning the adult age range from 21 to 80 years. We analyzed associations between medication-related anticholinergic burden and structural MRI volumes from participants (*n* = 3087, 52.2% female) of the population-based “Study of Health in Pomerania” (SHIP). Anticholinergic burden was obtained from the current medication plan using the Anticholinergic Burden Scale (ACB). All analyses were adjusted for age, sex, education, and total intracranial volume. We found statistically significant associations between the ACB and the left and right hippocampus volume but not for the basal forebrain cholinergic system. Complementary voxel-based analysis across all participants revealed FWE-corrected (*p* =  < 0.05) clusters in the temporo-parietal regions reaching into frontal areas, showing reduced volumes with higher ACB scores. We identified an association between anticholinergic burden of medication on hippocampal volume suggesting a potential inverse effect of such medication. This association highlights the importance of a careful prescription of medication with anticholinergic activity at any adult age.

**Supplementary Information:**

The online version contains supplementary material available at 10.1007/s11357-021-00497-w.

## Introduction

### Anticholinergic burden and cognition

Anticholinergic burden can be caused by medications with anticholinergic side effects. Particularly, older adults are at risk to receive potentially inappropriate medication with anticholinergic properties. At the same time, these people have a high risk of adverse events which can lead to physical and cognitive impairment. Previous studies showed an association between the intake of medication with anticholinergic properties and lower cognitive functioning in aging adult populations living at home or in institutional care [1, 2]. In a community-based sample, older adults with higher anticholinergic burden were cognitively impaired and had an increase of serum anticholinergic activity (SAA) as measured by a radioreceptor assay. SAA levels were significantly associated with lower scoring in the Mini Mental Status Examination [3].

A recent meta-analysis including 26 studies found inverse effects of anticholinergic burden on cognition and a higher risk of cognitive decline and dementia for people with high anticholinergic burden compared to people with no or low burden [4]. Additionally, a case–control study with more than 250,000 datasets from patients aged 55 years and above found that the risk of dementia increased by nearly 50% in patients with high anticholinergic burden compared to those with no to low anticholinergic burden over a 10-year period [5].

Few studies investigated the effects of anticholinergic burden on brain structures. One study indicated an association between higher anticholinergic burden and lower cortical thickness of the temporal lobe [6]. Results of the Baltimore Longitudinal Study of Aging including data of 723 individuals showed that participants with high anticholinergic burden had an increased rate of global and regional atrophy over a mean follow-up time of 20.1 years [7].

### Cholinergic neurons and dementia

For large parts of the cerebral cortex, the hippocampus, and thalamus, cholinergic input stems from the cholinergic basal forebrain (BF) [8]. The cholinergic input of the BF plays a major role in working memory, executive function, and attention [9]. The degeneration of cholinergic neurons in the BF occurs early and leads to a cholinergic deficit, which is hypothesized to be the main contribution to cognitive decline in AD, the most frequent cause of dementia in high age [10–12]. Studies from our group showed that the BF might already be affected in the pre-dementia stages of AD with different vulnerabilities in the subregions of the BF [13, 14]. A recent study found an increased risk in cognitive healthy older adults with anticholinergic medication for incident mild cognitive impairment. This risk was even higher in participants with Alzheimer’s disease (AD) risk genes and positive AD biomarkers in cerebrospinal fluid underlining the potential link between anticholinergic burden and AD.

To further analyze associations between the anticholinergic burden and structural brain parameters, we examined the data from the population-based Study of Health in Pomerania (SHIP). SHIP covers almost the entire adult age range with participants aged 20 to 90 years. As far as we know, this is the first examination of anticholinergic burden and brain structure in a population-based study.

### Hypothesis

We expected to find an inverse association between the anticholinergic burden of medication and brain structure. In this study, we combined the volumetric measurements of the basal forebrain as cholinergic output and the hippocampus as cholinergic input region and their high involvement in cognitive performance as hypothesis-driven approach and a regionally unbiased voxel-based morphometry to identify associations in any brain region.

## Material and methods

### Study population

The target population comprised adult German residents (20–79 years of age in 1997–2001) in northeastern Germany living in preselected cities (*n* = 3) and communities (*n* = 29) of Western Pomerania with a total population of 212,157. Within these communities, a random sample stratified by sex and age was drawn from the regional residence registries compromising 6267 eligible residents, of which 4308 participated in SHIP-START-0. Follow-up examination (SHIP-START-1) was conducted 5 years after baseline (2002–2006) and included 3300 subjects. From 2008 to 2012, the third phase of data collection (SHIP-START-2, *n* = 2333) was carried out including whole-body magnetic resonance imaging (MRI) sampling. Concurrent with SHIP-START-2 from 2008 to 2012, an independent age- and sex-stratified, random sample called SHIP-TREND-0 of 10,000 individuals (net sample size of 8826) was facilitated by centralization of local population registries in the Federal State of Mecklenburg-West Pomerania and out of these, 4420 (2275 women) people participated (response = 50.1%). More details on the study design, recruitment, and procedures have been published elsewhere [15]. Although not being part of the sample selection process, almost all participants are Caucasian due to the locally low rate of racial diversity.

All participants gave written informed consent and ethical approval was granted by the Ethics the Committee of the University of Greifswald. All study procedures have been in accordance with the 1964 Declaration of Helsinki and its later amendments.

Our analysis included data from two independent observational population studies including the SHIP-START-2 study and the SHIP-TREND-0 study. Subjects from SHIP-START-2 and SHIP-TREND-0 were asked to participate in an MRI assessment. After exclusion of subjects who refused participation or fulfilled exclusion criteria for the MRI assessment, 1163 individuals from SHIP-START-2 and 2154 individuals from SHIP-TREND-0 underwent the MRI scanning. Demographic characteristics of the study population can be found in Table 1, the age distribution in the supplemental figures.

**Table 1:** Characteristics of the study sample by cohort

	**Total sample**	**SHIP-START-2**	**SHIP-TREND-0**
*n*	3087	1066	2021
Age (in years)	52.15 (13.49)	55.05 (12.43)	50.63 (13.78)
Age range (minimum–maximum)	21–90	30–90	21–82
Gender (M/F)	1475/1612	504/562	971/1050
Education (< 10/ = 10/ > 10 years in school)	495/1723/869	213/602/251	282/1121/618
Smoking (none, ex-smoker, current smoker)^*^	1220/1163/701	421/432/210	799/731/491
Alcohol intake (in g/day)^*^	8.83 (12.57)	10.06 (13.48)	8.19 (12.03)
BMI (in kg/m^2^)^*^	27.55 (4.42)	27.56 (4.44)	27.54 (4.42)
Obesity (yes, no)^*^	845/2240	284/780	561/1460
Hypertension (yes, no)^*^	1465/1616	575/491	890/1125
Diabetes (yes, no)^*^	198/2886	82/983	116/1903
Lifetime MDD (yes, no)^*^	506/2402	146/765	360/1637
ACB	0.21 (0.70)	0.24 (0.77)	0.19 (0.66)
TIV (in cm^3^)	1464.02 (148.91)	1460.73 (150.03)	1465.76 (148.32)
Hippocampus L (in mm^3^)	2889.47 (308.32)	2866.76 (312.27)	2901.45 (305.62)
Hippocampus R (in mm^3^)	2955.79 (332.09)	2929.92 (342.62)	2969.43 (325.66)
Basal forebrain (in mm^3^)	674.09 (65.48)	667.58 (63.67)	677.53 (66.17)

Entries are mean (standard deviation) for continuous variables and absolute frequencies for categorical variables

Abbreviation: *BMI*, body mass index; *MDD*, major depressive disorder; *ACB*, Anticholinergic Cognitive Burden Scale sum score; *TIV*, total intracranial volume

^*^Information is not available for all individuals of the analysis sample and absolute frequencies for categorical variables

### MRI acquisition

All examinations were performed with one 1.5 T MRI scanner (Magnetom Avanto; Siemens Medical Systems, Erlangen, Germany) at the University Medicine Greifswald. From the total time of 90 min, the scanning time for the brain specific sequences took 10 min. For the volumetric and voxel-based analysis, we used an axial T1 MPRAGE (TR 19000 ms, TE 890 ms, flip angle 15°, voxel-size 1.0 × 1.0 × 1.0mm^3^). The complete protocol is published elsewhere [16]. Two radiologists with at least 5 years of experience documented known or accidental findings.

**Fig. 1:**
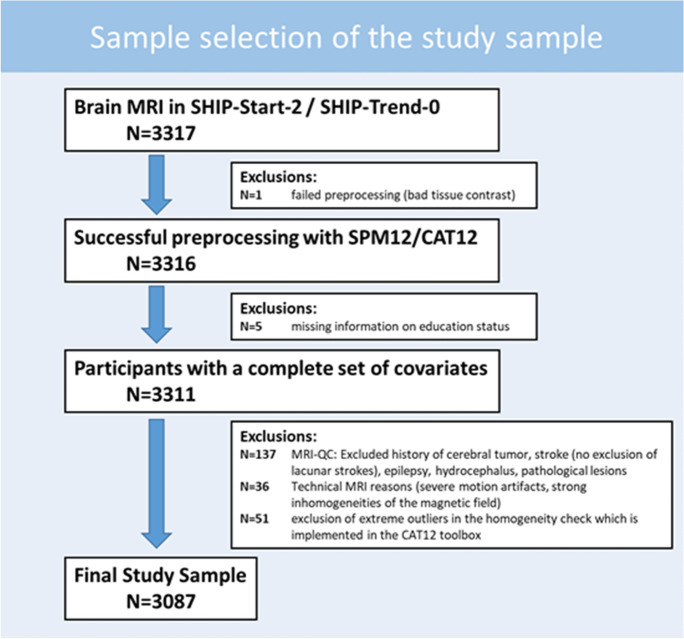
Flowchart sample selection

Participants with diagnosed epilepsy, territorial or sub-territorial post-ischemic lesions, or arachnoid cysts in frontal, parietal, or temporal regions were excluded from our analysis (details on the sample selection can be found in Fig. 1).

### MRI processing

The Statistical Parametric Mapping package (SPM12, Wellcome Trust Centre for Neuroimaging, University College London) and the Computational Anatomy Toolbox (CAT12) toolbox (developed by Christian Gaser, University of Jena, Germany, http://www.neuro.uni-jena.de) were used for processing and analysis. Firstly, we segmented images into gray matter (GM), white matter, and cerebrospinal fluid partitions. Secondly, the warping parameters from a high dimensional registration to Montreal Neurological Institute (MNI) standard space were applied to individual GM maps. Thirdly, GM-voxel values were modulated for non-linear warping and affine transformations and smoothed by a Gaussian kernel of 8 mm FWHM. Total intracranial volume was calculated as the sum of the total segmented GM, white matter, and cerebrospinal fluid volumes.

Individual GM volumes of BF and hippocampus were extracted automatically by summing up the modulated GM-voxel values within the respective region of interest (ROI) masks in the template spaces. The ROI masks of the BF and the hippocampus were used to assess the regional volume. The ROI masks of the BF were derived from histological and post-mortem MRI mapping of the BF and its subregions [14]. The hippocampus ROI was based on a manual delineation of the hippocampus in MNI standard space following the international harmonized standards [17] using an automated method [18].

### Assessment of medication

Individual medication was assessed by a computer-assisted face-to-face-interview at the study center. We identified the active ingredient and assigned a code from the Anatomical Therapeutic Chemical Classification System (ATC). The current version of the ATC was used at the time of data acquisition.

In a further step, we scored substances listed in the Anticholinergic Cognitive Burden Scale (see below). Substances not listed received a score of 0. The ACB medications do not include topical and inhaled products.

### Covariates

The SHIP study has participants over a wide age range, so we include age as cofounder into our analysis and sex, as a difference between male and female organisms and medication intake and comorbidities is presumable. As proxy for cognitive reserve, we added years of education into the model. A complimentary analysis (data shown in the supplement) included further covariates addressing vascular risk factors (obesity, smoking, arterial hypertension, and diabetes), alcohol, and lifetime diagnosis of depression into the model.

### Anticholinergic Cognitive Burden Scale

The ACB sum score uses a 1 to 3 scoring differentiating between evidence of anticholinergic activity from in vitro data (= 1); from literature, prescription information, and experts (= 2); and the potential to cause delirium in literature information, prescription information, or expert opinion (= 3) to classify the anticholinergic burden. The substance selecting strategy for the ACB was a Medline search for studies measuring the anticholinergic potential and cognitive effects of any drug [19]. In our study, we used the 2012 update of the ACB [20]. Only the medication taken regularly was included in the calculation of the ACB sum score; PRN (pro re nata—“as needed”) medication was skipped as only medication in current use was documented. Substances and the corresponding ATC codes are listed in supplemental Table 1.

### Cognitive assessments

The Auditory Verbal Learning and Memory Test (VLMT) [21] was completed as an optional test module in the SHIP study and was usually performed between the SHIP-START-1 and SHIP-START-2 examinations (*n* = 861). Parts of the Nurnberg Age Inventory (NAI) were included in the SHIP-TREND baseline visit. Verbal memory was assessed by immediate recall (NAI1, *n* = 2021) and late recall with distractor words (NAI2, *n* = 2006) after 20 min [22].

### Statistical analyses

We examined the sum score of the ACB with the volume of the hippocampus (left and right) and the basal forebrain (both hemispheres combined) in three separate linear regression models controlling for sex (male, female), age (modeled continuously using restricted cubic splines), education (< 10; = 10; > 10 years in school), cohort (SHIP-START-2, SHIP-TREND-0), and total intracranial volume in the statistics software R version 3.6.1 (https://www.r-project.org/). The statistical threshold of significance was defined to be *p* < 0.05 (uncorrected) and < 0.05/3 (after Bonferroni correction for multiple comparison).

For voxel-based morphometry (VBM) analyses, we used SPM12 to analyze the preprocessed GM segments. For the anticholinergic burden score, we conducted a linear regression model with the same set of covariates (sex (male, female), age (modeled continuously using restricted cubic splines), education (< 10, = 10, > 10 years in school), cohort (SHIP-START-2, SHIP-TREND-0), and total intracranial volume). We used the Masking Toolbox to define explicit masks to limit the number of voxels entering the VBM analyses on GM [23]. Specifically, we used the MATLAB script “make_majority_mask.m” to generate a gray matter mask with an absolute threshold of 0.2 and a consensus fraction of 99%.

The statistical threshold for significant voxels was set to a family-wise error (FWE)–corrected peak-level *p*-values p_peak,FWE_ < 0.05. The labeling of the significant clusters was done within the xjview toolbox (http://www.alivelearn.net/xjview) on the basis of the Anatomical Automatic Labeling atlas (AAL) [24]. In addition, we report clusters with a FWE-corrected cluster-level *p*-value p_cluster,FWE_ < 0.05.

Further, we extracted GM volumes of significant clusters which emerged in the VBM analysis to study the association with cognitive measures. As SHIP-START-2 and SHIP-TREND-0 had different cognitive parameters, the linear regression models were conducted separately in the two cohorts. The analyses were controlled for sex (male, female), age (modeled continuously using restricted cubic splines), education (< 10; = 10; > 10 years in school), and total intracranial volume.

The analysis of the cognitive parameters had only explorative character as cognitive measures were not available from all participants, different tests were used in SHIP-START-2 and SHIP-TREND-0 and not all assessments were performed at the time point of the medication assessment and MRI. The linear regression models were conducted separately in the two cohorts.

## Results

After excluding datasets due to technical reasons (MRI) or missing data, the analyses included 3087 individuals aged 21 to 90 years (mean = 52.15, SD = 13.49). Further sample characteristics stratified by the cohort are provided in Table 1.

In a first step, we performed a sensitivity analysis and checked whether associations were driven by outliers. Therefore, we excluded volumes of the left and right hippocampus and the basal forebrain which exceeded the range of three standard deviations from the mean. This left us with *n* = 3075 out of *n* = 3317 individuals in all three additional sensitivity analyses.

Out of 6289 medications reported, we identified 540 to be anticholinergic according to the ACB scale listing and 435 medications from these were regularly taken by 362 participants. The volumetric analysis (Table 2) showed a statistically significant inverse association between the ACB sum score and the volumes of the hippocampus (left *β* =  − 17.00, *p* = 1.28*10^−3^; right *β* =  − 20.93, *p* = 4.05*10^−4^). The ACB sum score and the BF volume were inversely associated as well (*β* =  − 1.82, *p* = 0.044), but the association did not reach significance after the Bonferroni correction for multiple comparisons.

**Table 2:** Results for the association of ROI volumes and ACB for chronical medication (*n* = 3087, inverse association)

ROI	Effect size *β*	SE	*t*	*p* (1-sided)
Hippocampus L	− 17.00	5.63	− 3.02	1.28*10^**−3**^
Hippocampus R	− 20.93	6.24	− 3.35	4.05*10^**−4**^
Basal forebrain	− 1.82	1.07	− 1.70	0.044

These results were stable and were not influenced by outliers.

**Fig. 2:**
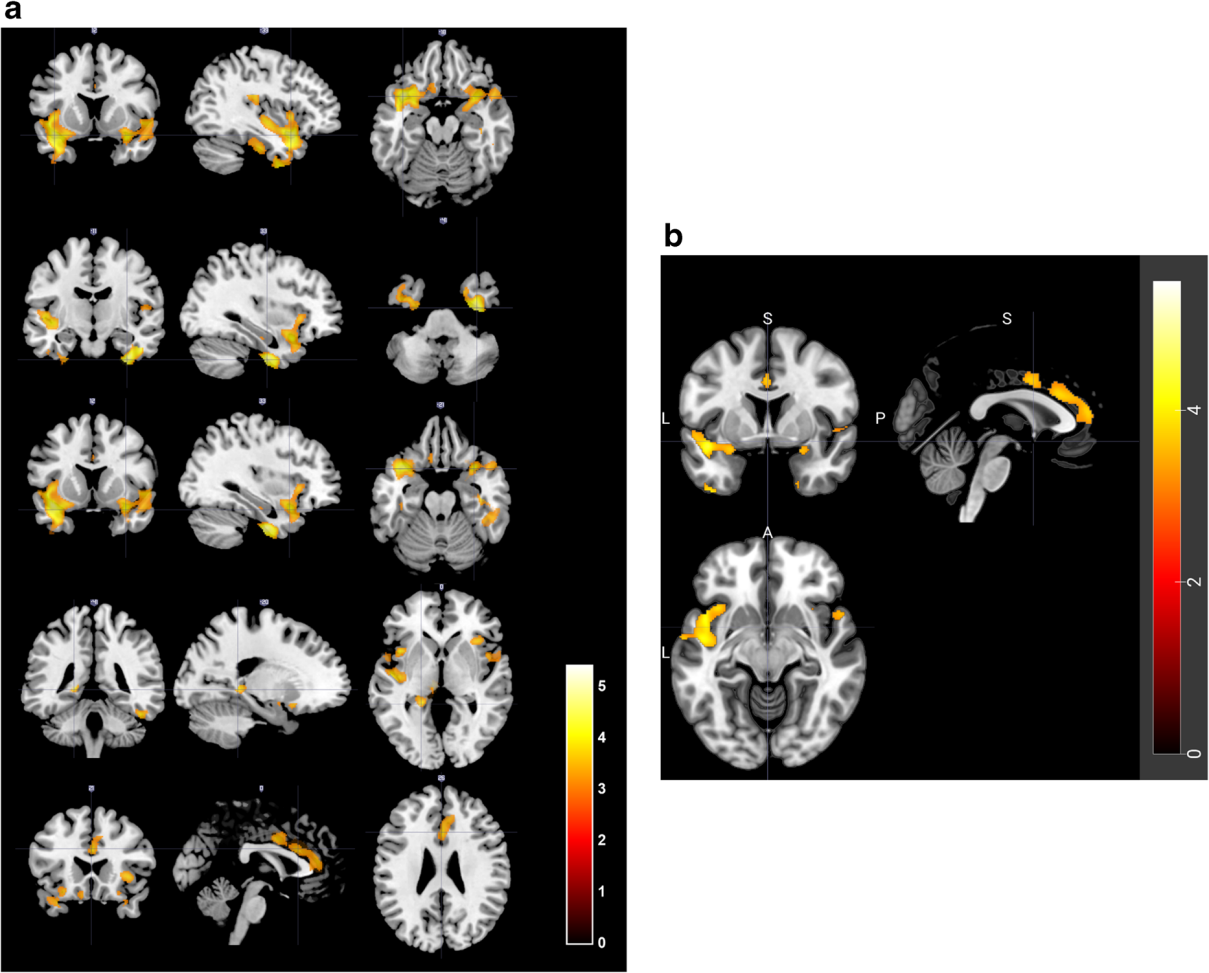
**a** VBM results for ACB for chronical medication (inverse association). The VBM analyses revealed five statistical significant clusters (> 500 voxels, FWE-corrected cluster-level *p*-value p_cluster,FWE_ < 0.05) that are negatively associated with the anticholinergic burden measured by the ACB score for chronical medication. **b** VBM results for ACB for chronical medication (inverse association). Rostral parts of the right hippocampus are included in the five statistical significant clusters (> 500 voxels, FWE-corrected cluster-level *p*-value p_cluster,FWE_ < 0.05) that are negatively associated with the anticholinergic burden measured by the ACB score for chronical medication

The VBM analyses (Fig. 2 and Table 3) revealed five statistical significant clusters (FWE-corrected cluster-level *p*-value, p_cluster,FWE_ < 0.05) that were inversely associated with anticholinergic burden measured by the ACB for regularly taken medication. Further, we found three smaller clusters of voxels that were significant on the FWE-corrected peak-level *p*-value (p_peak,FWE_ < 0.05). For detailed results, please see Table 3.

**Table 3:** VBM results for ACB for chronical medication (inverse association)

Cluster size (in voxels)	AAL-regions	Brodmann areas	p_cluster,FWE_	p_peak,FWE_	*t *score	Cohen’s *D*	Stereotaxic coordinates (in mm)		
							*x*	*y*	*z*
6563	L temporal pole (superior gyrus), L superior temporal gyrus, L insula, L inferior temporal gyrus, L Rolandic operculum, L inferior frontal gyrus (orbital part), L temporal pole (middle gyrus), L fusiform gyrus, L Heschl gyrus, L middle temporal gyrus, L supramarginal gyrus, L gyrus rectus, L superior frontal gyrus (orbital part), L amygdala, L olfactory cortex, L postcentral gyrus, L inferior frontal gyrus (opercular part)	38, 13, 47, 20, 22, 21, 41, 40, 34, 11, 36, 25, 28, 43	3.15*10^**−8**^	**0.007**	4.88	0.18	− 39	12	− 18
1854	R fusiform gyrus, R inferior temporal gyrus, R temporal pole (middle gyrus), R hippocampus, R parahippocampal gyrus, R cerebellum Crus1	20, 37, 36, 38, 21	1.34*10^**−3**^	**0.007**	4.88	0.18	33	− 11	− 41
2299	R temporal pole (superior gyrus), R insula, R amygdala, R Rolandic operculum, R inferior frontal gyrus (orbital part), R temporal pole (middle gyrus), R superior temporal gyrus, R Heschl gyrus, R olfactory cortex, R superior frontal gyrus (orbital part), R gyrus rectus, R hippocampus, R putamen, R parahippocampal gyrus, R inferior frontal gyrus (triangular part)	38, 47, 22, 13, 34, 43, 28, 6, 21	3.88*10^**−4**^	0.073	4.33	0.16	33	12	− 21
822	L thalamus, L hippocampus, L parahippocampal gyrus, L lingual gyrus, L precuneus	27, 30	**0.038**	0.202	4.04	0.15	− 20	− 41	0
1655	R anterior cingulate cortex, L anterior cingulate cortex, R middle cingulate cortex, L middle cingulate cortex	32, 24, 9, 33, 10	**0.002**	0.361	3.84	0.14	0	21	26

Further, we observe three smaller clusters of voxels that are statistically significant on the FWE-corrected peak-level *p*-value (p_peak,FWE_ < 0.05). Two of them are located within the first cluster of this table (133 voxels with peak coordinate [− 39, 12, − 18] comprising regions of the left temporal pole, insula, superior temporal, and inferior frontal gyrus and 24 voxels with peak coordinate [− 47, − 24, 17] situated in the left Rolandic operculum and supramarginal gyrus). The remaining one is located in the second cluster of the table (184 voxels with peak coordinate [33, − 11, − 41] comprising regions of the right fusiform and the inferior temporal gyrus)

*Abbreviations*: *AAL*, Anatomical Automatic Labeling; *FWE*, family-wise error; *L*, left hemisphere; *R,* right hemisphere, *VBM*, voxel-based morphometry

In the exploratory analysis of the cognitive assessments and the anticholinergic burden, ACB sum score had a statistically significant inverse association with VLMT (*p* = 0.038, effect size *β* =  − 0.25), NAI1 (*p* = 3.61*10^−6^, effect size *β* =  − 0.18) and NAI2 (*p* = 6.78*10^−3^, effect size *β* =  − 0.14).

## Discussion

In this population-based study across the adult age range, we found a statistically significant inverse association between the ACB sum score and the hippocampus volume. A statistically significant association between anticholinergic burden and the basal forebrain was evident only before multiple comparison correction.

Research on structural brain parameters and the association with anticholinergic activity of medication is still rare. Risacher and colleagues (2016) compared brain structures and anticholinergic medication of 451 cognitively healthy older adults in their cross-sectional study using data from the Alzheimer’s Disease Neuroimaging Initiative (ADNI) and the Indiana Memory and Aging Study (IMAS). They categorized the participants into an ACB-positive and an ACB-negative group. Participants taking medications with an ACB score of 1 (possible anticholinergic activity) were still included in the ACB-negative group. The total cortical volume and the total cortical thickness were reduced in the ACB-positive group compared to the ACB-negative group. Furthermore, they found an increased volume of the inferior lateral ventricles in the ACB-positive group as an indirect indicator for temporal-mesial brain atrophy in the positive group compared to ACB-negative participants [6]. Our VBM analysis confirmed an impact particularly on the temporo-mesial region containing the biggest cluster of inverse association with the ACB sum score in this region. The volumetric analysis of the hippocampus formation also revealed a reduced volume for the participants with higher ACB sum scores.

In a longitudinal study, Chuang et al. used the data from 723 participants of the Baltimore Longitudinal Study of Aging to compare brain structure and mid-life use of medication with anticholinergic potential. Over a mean follow-up time of 20.1 years, they found a greater rate of cortical atrophy in participants with medication scored 1 in the ACB compared to participants who did not take ACB-listed medication. The areas with higher atrophy rates were the right posterior cingulum, middle frontal, and left superior temporal gyrus. Our findings support this as both regions are part of the largest clusters we found in our voxel-based analysis. The cluster with the highest effect rates included large regions of the left temporal gyrus, inferior parts of the frontal gyrus, insula, and operculum as well as the supramarginal gyrus. Interestingly and in contrast to the study from Risacher and colleagues [6], Chuang’s group [7] did not find any statistically significant association between brain atrophy and ACB score in participants with higher ACB scores (scores 2 and 3). They argue that the heterogeneity of the group with higher ACB scores and the shorter time period of intake might be the reason why this group did not show any statistically significant associations with structural changes.

The pathomechanism behind these structural changes is still unclear. Cholinergic stimuli are suspected to be involved in mechanisms of neuroplasticity. Studies examining loss of cholinergic input in the hippocampus suggested a reduced neuroplasticity in this region due to a reduced ability to induce long-term potentiation, a mechanism involved in memory, learning, and neuroplasticity [25, 26]. Furthermore, neurogenesis in the hippocampus is decreased if cholinergic innervation is reduced [27, 28]. In addition, neurotrophic factors are also influenced by cholinergic innervation and are likely reduced through anticholinergic medication. Kotani et al. [29] showed that scopolamine, a substance with strong anticholinergic potential, reduced brain-derived neurotrophic factor (BDNF) in the hippocampus and cell processes needed for neuronal survival, namely the phosphorylation of cAMP response element binding protein (CREB) [29, 30]. Further studies are needed to better understand the effect of anticholinergic burden on a cell or subcellular level.

Interestingly, brain areas affected by anticholinergic burden in our and previous studies are, to a large extent, also brain regions affected in AD pathology.

The cholinergic hypothesis of AD indicates that the pathophysiological changes in the course of AD lead to a reduction of cholinergic input to the hippocampus and the neocortex [10, 31, 32]. A prospective study showed that partial restoration of the cholinergic deficit in AD has effects on structural parameters [33]. The authors used structural MRI data from 52 AD patients under medication with donepezil and 93 AD patients who did not take donepezil (data from patients before 1999, when donepezil was introduced in Japan). The group with donepezil medication showed a smaller reduction of hippocampal volume than controls. Similar results have been shown in the Hippocampus Study, a multicenter study with 332 participants with prodromal AD who received 10 mg of donepezil per day or placebo over 12 months with MRI imaging at baseline and at the end of treatment. A 45% reduction of atrophy rate in the annualized percentage change of hippocampal volume was detected in the group with donepezil compared to controls [34]. In addition, in another subgroup analysis (inclusion criterion was a sufficiently high quality of imaging for BF volumetry), participants with donepezil showed a lower rate of atrophy of the BF compared to placebo after treatment [35].

In an animal study with APP/PS1 transgenic mice (a common mouse model for AD) [36], researchers showed that the denervation of the BF and, consequently, the cutoff from cholinergic innervation was followed by a rapid beta-amyloid deposition, the hallmark neuropathological marker for AD [37]. A possible association between the intake of medication with anticholinergic properties and the course or incidence of pathophysiological processes is still lacking confirmation in human studies. In a community-based study assessing neuropathological findings at autopsy and medication of 420 non-geriatric and non-cognitively impaired participants, Gray and colleagues did not find any significant association between anticholinergic burden and post-mortem evidence of AD pathology [38]. However, they only identified medication with strong anticholinergic activity and calculated the total standard daily dosage, a method which multiplies the number of pills by the ratio individual dosage/minimum recommended dosage, in a 10-year window before a retrospectively identified date of onset of dementia. In this clinico-pathological study, AD pathology did not differ between participants with high, low, or no anticholinergic burden at autopsy. The effect of anticholinergic burden on the brain and on emerging neurodegeneration might not be reflected by the degree of amyloid or tau pathology but by neuronal and synaptic loss. Our study results might reflect this loss rather than the underlying neurodegenerative pathology, as a reduction in neuronal or synaptic densities leading to structural changes that can be measured using the volumetric analyses.

Hypothetically, if regions typically affected in AD are presumably also sensitive to changes in cholinergic input, one would expect to see an effect of anticholinergic burden in the basal forebrain. However, we did not find a statistically significant association between the basal forebrain volume and the ACB sum score after multiple comparison correction. However, there was a trend towards significance. This finding is similar to the results from a neuropathological study on brains of 298 donors. Richardson and colleagues found an association between anticholinergic medication and neuronal loss in the BF, but the effect did not reach significance after the correction for multiple comparison. In addition, they did not observe any statistically significant interaction between the prevalence of AD pathology and anticholinergic medication [39]. Using data from the Alzheimer’s Disease Neuroimaging Initiative (*n* = 64), a clinic-pathological association study found BF atrophy in antemortem MRI associated with Thal amyloid phases and the presence of Lewy body pathology in post-mortem neuropathological assessments. These results did not reach significance after false discovery rate (FDE) [40]. The effects of anticholinergic burden on the emergence, progression, and type of brain pathology as well as factors of resilience remain unclear and may include further yet unknown mechanisms like a direct suppression of cholinergic input by anticholinergic medication or a (maybe indirect) upregulation of cholinergic activity in the BF. Mufson et al. suggest that new sprouting of cholinergic terminals of the BF toward the hippocampus might be stimulated in the MCI stage to compensate for the reduced entorhinal glutamergic input in the hippocampus [41]. Whereas the BF reacts by increasing its function, the hippocampus might not have this feedback mechanism from anticholinergic stress. Indeed, the BF is the main source of cholinergic innervation for the hippocampus region but the neurotransmitter release of these cholinergic neurons of the BF is regulated by adenosine and is, therefore, not directly affected by a potential disbalance of acetylcholine.

The exploratory analysis of the cognitive assessments revealed a statistically significant inverse association between cognitive test parameters and the ACB sum score underlining the potential clinical significance of the findings in our study. The impact of anticholinergic burden on cognition has been described in several studies [1–3] and our results support these findings. However, in the mediation analysis between the ACB and the score of the VLMT or NAI with hippocampal volume as a mediator, the mediation effect in this cross-sectional analysis was not significant (results not shown) suggesting a more complex interaction between anticholinergic medication, brain structure, and cognitive function. Further and particularly longitudinal studies are needed to gain more insides of these mechanisms and potential interactions.

### Limitations

Volumetric measures have limitations and volume loss in MRI analysis does not necessarily indicate cellular loss. An upregulation of the individual BF cells could possibly go along with an increase of the cell body. The volume remains stable; however, the number of cells decreases.

Although we were not able to combine the cognitive assessment data from both cohorts because different tests were used, the data from each cohort was sufficient to find a significant correlation showing the clinical relevance of our research question.

In this study, as in many pharmaco-epidemiological studies, the identified associations between anticholinergic burden and brain structures do not allow a distinction between effect of medication, effect of indication of medication, or even a third, yet unknown, joined factor. All 99 active ingredients listed in the ACB share the anticholinergic activity with a wide distribution over several different indications. In an additional analysis (data shown in the supplement table S3), we included more covariates addressing vascular risk factors (obesity, smoking, arterial hypertension, and diabetes), alcohol, and lifetime diagnosis of depression into our model. This did not substantially change the results underlining the potential independent effect of anticholinergic burden on brain structure seen by this association.

The SHIP studies were conducted in a region of Germany with a low rate of ethnical diversity. Although not intended by sample selection, almost all participants in this study were Caucasian, which results in a limitation of the interpretation of our data in a trans ethnical context.

### Strengths

Compared to previous studies with restricted target groups (mostly geriatric patients), we were able to show an association between anticholinergic burden and brain structure in a representative, population-based cohort covering most of the adult age span. Furthermore, brain scans were performed in a single center on a single scanner ensuring consistency.

## Conclusion

The complex interaction of anticholinergic activity and the effect on brain structures is still not fully understood and might differ depending on pre-existing neuropathological conditions of the brain. The shown association indicates the potentially harmful effects of anticholinergic burden on brain structure and cognition highlighting the need for careful consideration when prescribing such medication. However, many questions are still not answered and further studies are needed to better understand the mechanisms of interaction.

## Supplementary Information

Below is the link to the electronic supplementary material.

Supplementary file1 (DOCX 101 KB)
