# Supplementary material for: Inverse association between the anticholinergic burden and hippocampus volume in a population-based cohort across the entire adult age range
Source: GeroScience. 2021 Dec 23;44(3):1715–26. doi: 10.1007/s11357-021-00497-w (PMC9213601; doi:10.1007/s11357-021-00497-w)
Supplement: Supplementary file 1 — Supplementary file1 (DOCX 101 KB) [file 11357_2021_497_MOESM1_ESM.docx]

**Supplemental Material**

**Overview**

Supplemental Figures and Tables:

Figure 1: Age distribution of the study sample

Supplemental Table 1: ACB Scale

Table of drugs, burden load, ATC Codes, Example calculation.

Supplemental Table 2:

Association of ROI volumes and ACB for chronical medication, adjustment for more covariates

Supplemental Table 3:

VBM results for ACB for chronical medication

**Supplemental Figures**


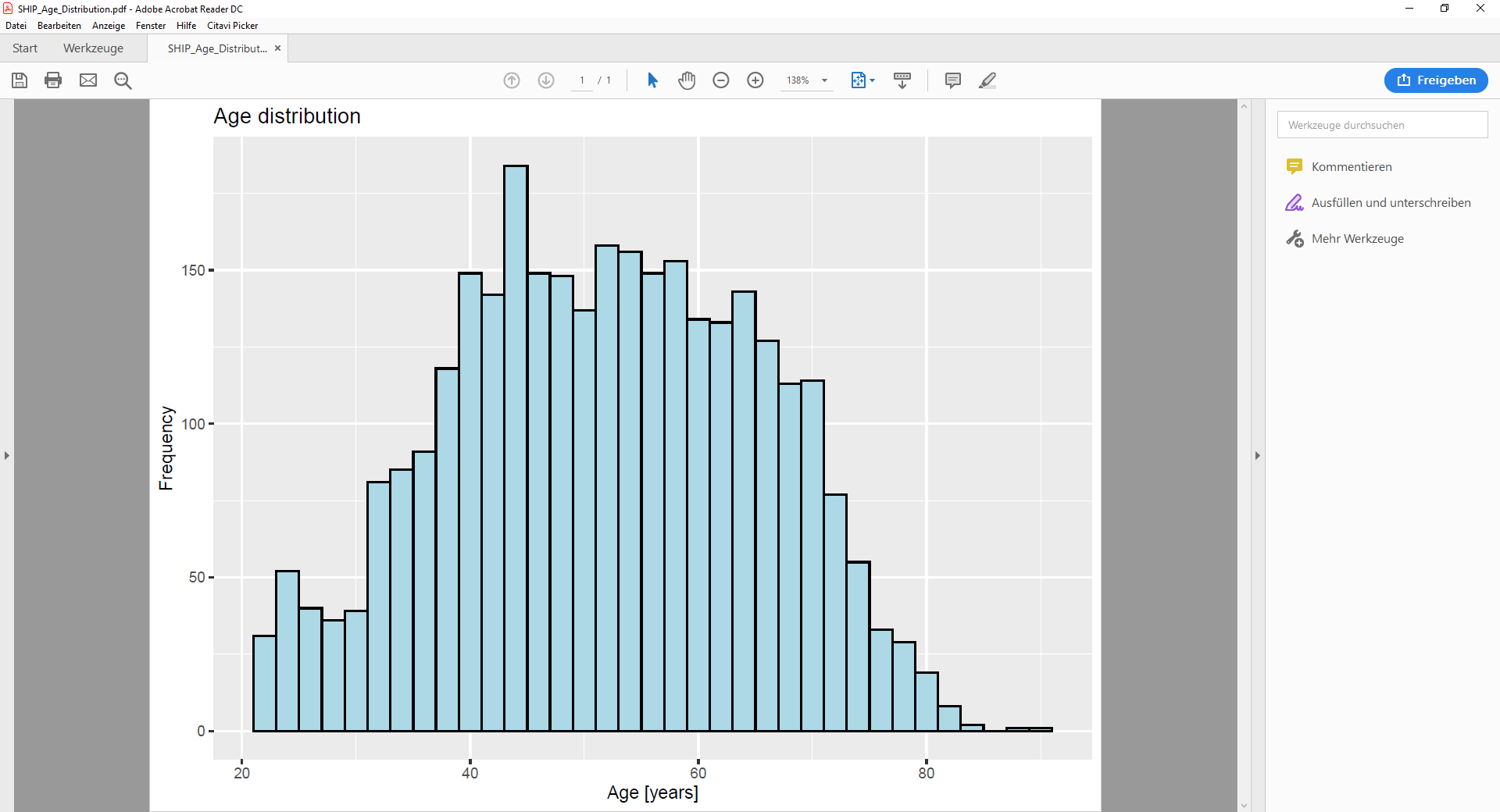


Supplemental figure 1: Age distribution of the study cohort.

**Supplemental Tables**

Supplemental Table S1: ACB Scale

Table of drugs, burden load, ATC Codes, Based on Aging Brain Care (2011) and the ATC lists from 2008 to 2012 to match the period of examination of the SHIP study.

| **Drug** | **Burden Load** | **Corresponding ATC Codes** |
| --- | --- | --- |
| Alimemazine | 1 | R06AD01 |
| Alprazolam | 1 | N05BA12 |
| Alverine | 1 | A03AX08, A03AX58 |
| Aripiprazole | 1 | N05AX12 |
| Asenapine | 1 | N05AH05 |
| Atenolol | 1 | C07AB03, C07AB11, C07BB03, C07CB03, C07CB53, C07DB01, C07FB03 |
| Bupropion | 1 | N06AX12, N07BA02 |
| Captopril | 1 | C09AA01, C09BA01 |
| Cetirizine | 1 | R06AE07, R06AE57 |
| Chlorthalidone | 1 | C03BA04, C03BB04, C03EA06 |
| Cimetidine | 1 | A02BA01, A02BA51 |
| Clidinium | 1 | A03CA02 |
| Clorazepate | 1 | N05BA05 |
| Codeine | 1 | N02AA08, N02AA58, N02AA59, N02AA64, N02AA65, N02AA66, N02AA69, N02AA79, N02CX58, R05DA04, R05DA12, R05DA14, R05DA54, R05DA64 |
| Colchicine | 1 | L01CC, M04AC01 |
| Desloratadine | 1 | R06AX27 |
| Diazepam | 1 | N05BA01, N05BA17 |
| Digoxin | 1 | C01AA02, C01AA05, C01AA08, C01AA52, C01AA55, C01AA58 |
| Dipyridamole | 1 | B01AC07, C01DX21, C01DX71 |
| Disopyramide | 1 | C01BA03 |
| Fentanyl | 1 | N01AH01, N01AH51, N02AB03 |
| Fluvoxamine | 1 | N06AB08 |
| Furosemide | 1 | C03CA01, C03CB01, C03EB01 |
| Haloperidol | 1 | N05AD01 |
| Hydralazine | 1 | C02DB01, C02DB02, C02LG01, C02LG02, C02LG51 |
| Hydrocortisone | 1 | A01AC03, A07EA02, C05AA01, C05AA51, D07AA02, D07AB02, D07AB11, D07AC16, D07BA04, D07BB04, D07CA01, D07CB06, D07XA01, H02AB09, R01AD60, S01BA02, S01BB01, S01BX01, S01CA03, S01CB03, S02BA01, S02CA03, S03CA04 |
| Iloperidone | 1 | N05AX14 |
| Isosorbide | 1 | C01DA08, C01DA14, C01DA58, C05AE02 |
| Levocetirizine | 1 | R06AE09 |
| Loperamide | 1 | A07DA03, A07DA05, A07DA53 |
| Loratadine | 1 | R06AX13 |
| Metoprolol | 1 | C07AB02, C07AB52, C07AB52, C07BB02, C07BB52, C07CB02, C07FB02, C07FB24 |
| Morphine | 1 | A07DA52, G04BE07, N02AA01, N02AA04, N02AA09, N02AA51, N02AA57, N02AF, N02AG01, N04BC07, R05DA01, R05DA05, S01XA06, V03AB45, V70AA02 |
| Nifedipine | 1 | C08CA05, C08CA55, C08GA01 |
| Paliperidone | 1 | N05AX13 |
| Prednisone | 1 | A01AC04, A01AC54, A07EA01, A07EA03, A07EA51, C05AA04, C05AA54, D07AA01, D07AA03, D07AC14, D07BA01, D07CA02, D07CA03, D07XA02, D10AA02, D10AA06, H02AB04, H02AB06, H02AB07, H02AB15, H02AB54, H02AB56, H02BX01, H02BX06, R01AD02, R01AD52, S01BA04, S01BB02, S01CA02, S01CA08, S01CA09, S01CA12, S01CB02, S02BA03, S02CA01, S03BA02, S03CA02 |
| Quinidine | 1 | C01BA01, C01BA51, C01BA71, C08DA81 |
| Ranitidine | 1 | A02BA02, A02BA07 |
| Risperidone | 1 | N05AX08 |
| Theophylline | 1 | C01EB21^*^, C01EB22, C01EX66, R03DA02, R03DA04, R03DA52, R03DA54, R03DA74, R03DB02, R03DB04 |
| Trazodone | 1 | N06AX05 |
| Triamterene | 1 | C03DB02, C03EA21 |
| Venlafaxine | 1 | N06AX16, N06AX23 |
| Warfarin | 1 | B01AA03 |
| Amantadine | 2 | J05AC04, N04BB01 |
| Belladonna | 2 | A03B, A03BA, A03BA04, A03BB, A03CB, A03CB02, A03DB, A06AB30 |
| Carbamazepine | 2 | N03AF01 |
| Cyclobenzaprine | 2 | M03BX08 |
| Cyproheptadine | 2 | A15AA01, A15AA51, R06AX02 |
| Loxapine | 2 | N05AH01 |
| Meperidine | 2 | N02AB02, N02AB52, N02AB72, N02AG03 |
| Methotrimeprazine | 2 | N05AA02 |
| Molindone | 2 | N05AE02 |
| Nefopam | 2 | N02BG06 |
| Oxcarbazepine | 2 | N03AF02 |
| Pimozide | 2 | N05AG02 |
| Amitriptyline | 3 | N06AA09, N06AA25, N06CA01 |
| Amoxapine | 3 | N06AA17 |
| Atropine | 3 | A02AG02, A03BA01, A03BB02, A03CB03, A03CB04, G04BD15, G04BD65, H01AC, H01AC01, N04AC30, S01FA01, S01FA05, S01FA51, V03AB44 |
| Benztropine | 3 | N04AC01 |
| Brompheniramine | 3 | R06AB01, R06AB06, R06AB51, R06AB56 |
| Carbinoxamine | 3 | R06AA08 |
| Chlorpheniramine | 3 | R06AB04, R06AB54 |
| Chlorpromazine | 3 | N05AA01 |
| Clemastine | 3 | D04AA14, R06AA04, R06AA54 |
| Clomipramine | 3 | N06AA04 |
| Clozapine | 3 | N05AH02 |
| Darifenacin | 3 | G04BD10 |
| Desipramine | 3 | N06AA01 |
| Dicyclomine | 3 | A03AA07, G04BD13, G04BD63 |
| Dimenhydrinate | 3 | A04AB02, A04AB52 |
| Diphenhydramine | 3 | A04AB05, A04AB55, D04AA32, D04AA33, D04AA82, N01BX06, N05CM20, N05CX07, R06AA02, R06AA52, S01GX16 |
| Doxepin | 3 | N06AA12 |
| Doxylamine | 3 | A04AB56, N05CM21, R06AA09, R06AA59 |
| Fesoterodine | 3 | G04BD11 |
| Flavoxate | 3 | G04BD02 |
| Hydroxyzine | 3 | N05BB01, N05BB51, R06AX32 |
| Hyoscyamine | 3 | A03BA03, A03CB31 |
| Imipramine | 3 | N06AA02, N06AA03 |
| Meclizine | 3 | A04AB04, A04AB54, R06AE05, R06AE55 |
| Methocarbamol | 3 | M03BA03, M03BA53, M03BA73 |
| Nortriptyline | 3 | N06AA10, N06CA06 |
| Olanzapine | 3 | N05AH03 |
| Orphenadrine | 3 | M03BC01, M03BC51, N04AB02 |
| Oxybutynin | 3 | G04BD04 |
| Paroxetine | 3 | N06AB05 |
| Perphenazine | 3 | N05AB03 |
| Promethazine | 3 | A04AB58, C05AX14, D04AA10, D04AA40, N05CM22, N05CX13, R06AD02, R06AD05, R06AD10, R06AD52, R06AD55 |
| Propantheline | 3 | A03AB05, A03CA34 |
| Propiverine | 3 | G04BD06 |
| Quetiapine | 3 | N05AH04 |
| Scopolamine | 3 | A03BB01, A03BB03, A03CB01, A03CB38, A03DB04, A03EA02, A04AD01, A04AD51, N05CM05, S01FA02, S01FA03 |
| Solifenacin | 3 | G04BD08 |
| Thioridazine | 3 | N05AC02 |
| Tolterodine | 3 | G04BD07 |
| Trifluoperazine | 3 | N05AB06 |
| Trihexyphenidyl | 3 | N04AA01 |
| Trimipramine | 3 | N06AA06 |
| Trospium | 3 | A03AB20, A03DA06, A03EA04, G04BD09, G04BD59 |
| **Combination drugs** |  |  |
| Furosemid und Triamteren | 2 | C03EB21 |
| Metoprolol und Nifedipin | 2 | C07FB22 |
| Atenolol und Nifedipin | 2 | C07FB23 |
| Prednisolon und Promethazin | 4 | V03AB05 |

^*^In 2008 C01EB21 codes theophylline. From 2009 on this code is used for regadenoson.

**Example for the calculation of the ACB:**

Participants medication plan includes acetylsalicylic acid (ACB score =0), Solifenacin (ACB score = 3) and Metoprolol (ACB score = 2). The ACB sum score for this participant is 5 (0+3+2=5).

**Supplementary table S2: Results for the association of ROI volumes and ACB for chronical medication (N=2,868, inverse association), adjustment for arterial hypertension, obesity, smoking, alcohol, life time diagnosis of depression**

| **ROI** | **Effect size β** | **SE** | **t** | **p (1-sided)** |
| --- | --- | --- | --- | --- |
| Hippocampus L | -15.49 | 5.77 | -2.68 | **7.34*10^-3^** |
| Hippocampus R | -19.65 | 6.40 | -3.07 | **2.17*10^-3^** |
| Basal forebrain | -1.64 | 1.10 | -1.49 | 0.135 |
| Total Gray Matter | -2.88 | 0.73 | -3.94 | **8.21*10^-5^** |
| Total White Matter | -0.27 | 0.80 | -0.34 | 0.73 |

Additional adjustments: Obesity: BMI <30, >=30; Smoking: none, ex, current; hypertension: yes, if systolic >=140 or diastolic >=90 or intake of antihypertensive medication; diabetes mellitus: yes, if HBA1C >=6.5 or intake of antidiabetic medication; alcohol: gram per day.

**Supplementary Table S3: VBM results for ACB for chronical medication (N=2,868), adjustment for more covariates (arterial hypertension, obesity, smoking, alcohol, life time diagnosis of depression)**

| **Cluster size** | **AAL-Regions** | **Brodman areas** | **p_cluster, FWE_** | **p_peak, FWE_** | **t score** | **Cohen’s D** | **Stereotaxic coordinates** [in mm] | | |
| --- | --- | --- | --- | --- | --- | --- | --- | --- | --- |
| [in voxels] |  |  |  |  |  |  | **x** | **y** | **z** |
| 5290 | L temporal pole (superior gyrus), L insula, L inferior temporal gyrus, L superior temporal gyrus, L temporal pole (middle gyrus), L fusiform gyrus, L inferior frontal gyrus (orbital part), L middle temporal gyrus, L amygdala, L superior frontal gyrus (orbital part), L olfactory cortex, L Heschl gyrus, L gyrus rectus | 38, 20, 47, 13, 21, 22, 34, 36, 28 | **2.09*10^-7^** | **0.041** | 4.49 | 0.17 | -39 | 12 | -18 |
| 1335 | R fusiform gyrus, R inferior temporal gyrus, R hippocampus, R parahippocampal gyrus | 20, 21, 36 | **0.005** | **0.010** | 4.83 | 0.18 | 33 | -11 | -41 |
| 1364 | R temporal pole (superior gyrus), R insula, R temporal pole (middle gyrus), R inferior frontal gyrus (orbital part), R amygdala, R olfactory cortex, R superior frontal gyrus (orbital part), R putamen, R hippocampus | 38, 47, 13, 22, 34, 28, 21 | **0.005** | 0.181 | 4.09 | 0.15 | 33 | 12 | -21 |
| 1053 | R anterior cingulate cortex, L anterior cingulate cortex, R middle cingulate cortex | 32, 24, 9, 10, 33 | **0.014** | 0.276 | 3.95 | 0.15 | 0 | 41 | 12 |

**Abbreviations:** AAL = Anatomical Automatic Labeling; FWE = family-wise error; L = left hemisphere; VBM= voxel-based morphometry.

Additional adjustments: Obesity: BMI <30, >=30; Smoking: none, ex, current; hypertension: yes, if systolic >=140 or diastolic >=90 or intake of antihypertensive medication; diabetes mellitus: yes, if HBA1C >=6.5 or intake of antidiabetic medication; alcohol: gram per day

Further, we observe two smaller clusters of voxels that are statistically significant on the FWE-corrected peak-level p-value (p_peak,FWE_ < 0.05). One of them is located within the first cluster of this table (4 voxels with peak coordinate [-39 12 -18] within the left temporal pole (superior gyrus). The other one is located in the second cluster of the table (125 voxels with peak coordinate [33 -11 -41] within the right fusiform).
